# Long Preservation of AAV-Transduced Fluorescence by a Modified Organic Solvent-Based Clearing Method

**DOI:** 10.3390/ijms23179637

**Published:** 2022-08-25

**Authors:** Tao Lu, Munehisa Shinozaki, Narihito Nagoshi, Masaya Nakamura, Hideyuki Okano

**Affiliations:** 1 Department of Physiology, Keio University School of Medicine, 35 Shinanomachi, Shinjuku-ku, Tokyo 160-8582, Japan; 2 Department of Orthopaedic Surgery, Keio University School of Medicine, 35 Shinanomachi, Shinjuku-ku, Tokyo 160-8582, Japan

**Keywords:** AAV, retrograde tracing, tissue clearing, endogenous fluorescence, light-sheet fluorescence microscopy

## Abstract

The development of tissue clearing technologies allows 3D imaging of whole tissues and organs, especially in studies of the central nervous system innervated throughout the body. Although the three-dimensional imaging of solvent-cleared organs (3DISCO) method provides a powerful clearing capacity and high transparency, the rapid quenching of endogenous fluorescence and peroxide removal process decreases its practicability. This study provides a modified method named tDISCO to solve these limitations. The tDISCO protocol can preserve AAV-transduced endogenous EGFP fluorescence for months and achieve high transparency in a fast and simple clearing process. In addition to the brain, tDISCO was applied to other organs and even hard bone tissue. tDISCO also enabled us to visualize the long projection neurons and axons with high resolution. This method provides a fast and simple clearing protocol for 3D visualization of the AAV- transduced long projection neurons throughout the brain and spinal cord.

## 1. Introduction

Three-dimensional (3D) investigation of the cellular morphology and structure in individual organs, such as comprehensive and long projection neurons in the central nervous system (CNS), has become essential in the biomedical field [1,2]. Tissue clearing methods for the adult rodent brain have been reported recently [3], including organic solvent-based (such as 3DISCO [4], FDISCO [5], FluoClearBABB [6], and uDISCO [7]), aqueous-based (such as CUBIC [8], Sca*l*eS [9], SeeDB [10], and PACT [11]) and hydrogel embedding (such as CLARITY [12] and SHEILD [13]) clearing methods. The improvement to achieve a high level of transparency, endogenous fluorescence signal, and time efficiency during the clearing process is still being challenged.

Among those three types of clearing methods, the organic solvent-based clearing method shows a rapid clearing procedure, high transparency, and tissue shrinkage [3,14,15,16], which accelerates the process of sample preparation, imaging, and image processing for large tissues, such as adult mouse brain. The clearing protocol called three-dimensional imaging of solvent-cleared organs (3DISCO) achieves a fast and powerful clearing performance using tetrahydrofuran (THF) and dibenzyl ether (DBE). However, the peroxides are easily formed in 3DISCO’s solutions, especially in DBE, which results in rapid degradation of endogenous fluorescence proteins within 48 h [17,18], such as EGFP (Enhanced Green Fluorescent Protein), during tissue clearing and storage stages. In order to address this issue, several strategies were reported. In the study of FDISCO [5], the endogenous GFP fluorescence can be preserved over months after the adjustment of pH and temperature. Stabilization of purified THF, DBE, or BABB also effectively preserves EGFP signals for more than a year [19]. However, the procedure to remove peroxides in THF and DBE solutions is necessary for these methods. The complex instruments and processes were used as previously reported [20], which limits their availability. In the protocol of FluoClearBABB [6], the pH of all solutions was adjusted to alkaline conditions (pH: 9.5). It used the 1-propanol or *tert*-butanol as dehydration solution and the BABB (the mixture of benzyl alcohol and benzyl benzoate) as refractive index matching (RI-matching) solution. Other methods based on *tert*-butanol, such as uDISCO and a-uDISCO, achieved GFP fluorescence preservation by adjusting pH, temperature, and RI-matching solution. However, the procedure using *tert*-butanol contains multiple dehydration steps and a longer clearing time than 3DISCO. Although there are methods to amplify fluorescent signals by immunostaining with brighter chemical fluorescence dyes [21,22,23], the procedure to achieve full chemical dye-penetration and labeling in whole organs remains a challenge.

In this study, we modified the protocol of 3DISCO in three aspects throughout the clearing procedure, named tDISCO: (1) replacing the process for removing peroxide in THF by reducing air exposure using a glass pipette for transferring solutions; (2) keeping alkaline pH in all clearing conditions; (3) replacement of DBE with alkaline BABB-D4 (pH 9.0–10.0). After these adjustments, tDISCO enables the preservation of endogenous fluorescence (EGFP and mCherry) labeled by AAV over months and high transparency in a faster clearing process. tDISCO could also be applied to various organs. Combined with light-sheet fluorescence microscopy and confocal microscope, tDISCO was applied for 3D imaging and reconstruction of long projection neuronal circuits with high resolution. This method provides a fast and simple protocol for 3D imaging of AAV-transduced long projection neurons, which can be further applied to analyze the whole-organ imaging with high-resolution in biomedical science fields.

## 2. Results

### 2.1. tDISCO Preserves Endogenous EGFP Fluorescence for Months

To optimize the procedure of the organic solvent-based clearing method, we tested the concentration of peroxide in THF and DBE solutions (1 L or 1 KG volume) after usage. We found that the concentration of peroxide in THF was at a low level (<0.5 mg/L) within one year, whereas peroxides were easily formed within months in DBE (2~5 mg/L) (Supplementary Figure S1). Therefore, the procedure for removing peroxide in THF was replaced with using glass pipettes for transferring solution in the tDISCO protocol (Supplementary Figure S2).

Previous studies reported that the GFP fluorescence could be better maintained in alkaline conditions in clearing procedures [5,14,24]. To retain the fast-clearing capacity and improve endogenous fluorescence preservation, we used alkaline THF solution (pH 9.0~10.0) in the dehydration and delipidation processes in the tDISCO protocol. Since the DBE can form peroxidase easily, which causes fluorescence quenching, it was replaced with a-BABB-D4 (alkaline pH of BABB-D4). BABB-D4 is the final RI-matching solution used in uDISCO, which allows more stabilization of GFP fluorescence than the DBE used in 3DISCO [7].

**Figure 1:**
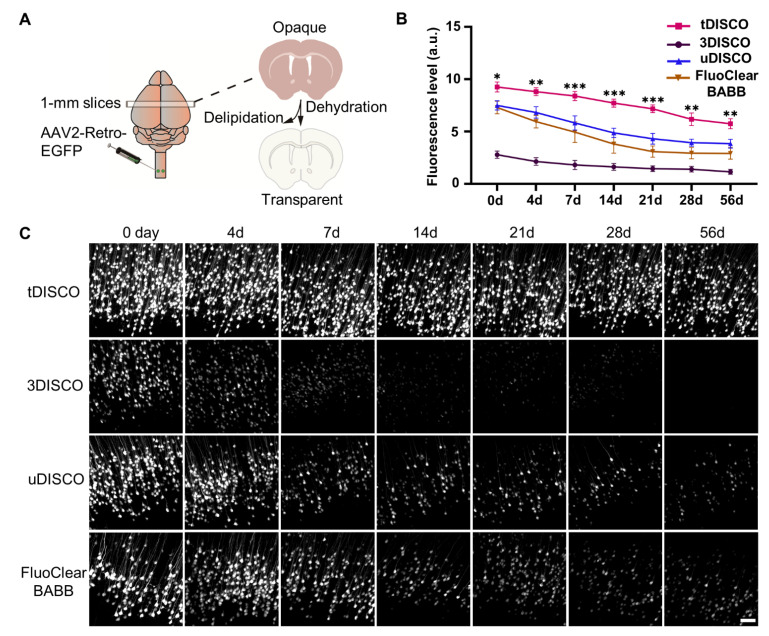
Comparison of the fluorescence preservation performance of tDISCO and other solvent-based clearing methods. AAV2-Retro-EGFP was injected bilaterally in the C4/5 segment. (**A**) The schema shows virus injection, brain slice preparation and clearing procedure. One-millimeter-thick brain slices were collected 2 w after injection. (**B**) Quantitative analysis of the fluorescence level (0 d~56 d) in each group after clearing. (**C**) Time course of fluorescent intensity after tDISCO clearing compared with 3DISCO, uDISCO, and FluoClearBABB clearing methods. All images are presented as maximum intensity projection (MIP) of z-stack (81 μm thickness, *n* = 3 slices per method). Scale bars: 50 μm. All data are shown as means ± SD. * *p* < 0.05, ** *p* < 0.01, and *** *p* < 0.001.

To examine the application of tDISCO for AAV-transduced EGFP fluorescence preservation, AAV2-Retro-EGFP was bilaterally injected into the cervical spinal cord. One-millimeter-thick brain slices were collected 2 w post-injection (Figure 1A). The clearing procedure for brain slices is described in Table 1. After simple dehydration and delipidation treatment, a sample became transparent after being immersed in RI-matching solutions. The cleared brain slices were mounted on slides before imaging. The EGFP^+^ neurons in the cortex were captured using a confocal microscope equipped with a 20× objective (NA, 0.8) at 0 to 56 days after clearing. Our strategy showed higher signals of endogenous EGFP fluorescence from 0 to 56 days after clearing compared with other solvent-based clearing methods (Figure 1B,C). We quantified the level of AAV-labeled EGFP fluorescence by tDISCO clearing over time compared with other representative solvent-based clearing methods. The level of fluorescence from cleared brain slices at day zero (tDISCO, 9.26 ± 0.42; uDISCO, 7.49 ± 0.39; FluoclearBABB, 7.28 ± 0.53; 3DISCO, 2.78 ± 0.31) showed that tDISCO improved the EGFP fluorescence by more than 20% (uDISCO and FluoclearBABB) and 200% (3DISCO). We also quantified the quenching rate at day 56 ((tDISCO, 37.9%; uDISCO, 48.7%; Fluoclear-BABB, 60.0%; 3DISCO, 58.7%), which suggested that tDISCO could preserve the endogenous fluorescence better. These data indicated that endogenous EGFP signals were preserved for months after tDISCO clearing, suggesting a more stable and long-term preservation capacity of tDISCO than the other three solvent-based clearing methods.

### 2.2. tDISCO Shows the Fast and Effective Clearing Performance on Adult Mouse Brain

**Figure 2:**
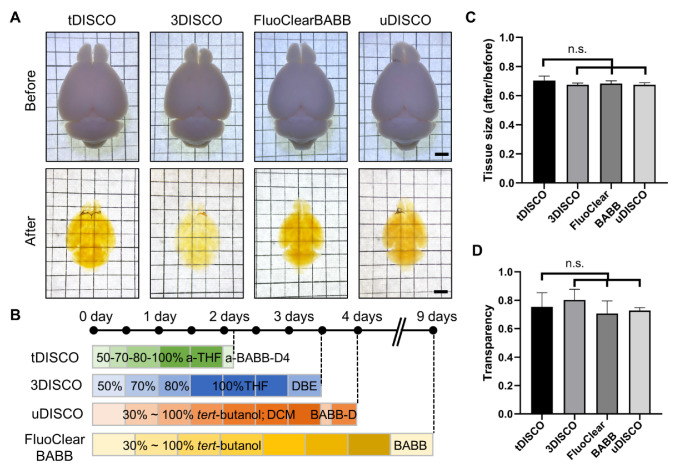
Comparison of the clearing performance on the adult whole brain of tDISCO and other solvent-based clearing methods. (**A**) Images of the whole brain before and after clearing. (**B**) The clearing processes and timelines for the adult whole brain by each clearing method. (**C**) The brain size change after tDISCO, 3DISCO, FluoClearBABB, and uDISCO clearing (*n* = 5 brains for each method). (**D**) Transparency of the whole brain after tDISCO, 3DISCO, FluoClearBABB, and uDISCO clearing (*n* = 5 brains for each method). Scale bars: 2 mm. All data are shown as means ± SD. n.s., not significant.

In order to test the clearing performance of tDISCO on the adult mouse brain, whole mouse brains were cleared using tDISCO, 3DISCO, FluoClearBABB, and uDISCO. The bright-field images for whole brains were captured before and after clearing. All cleared brains showed light brown/yellow color and tissue shrinkage (Figure 2A), consistent with the features of solvent-based clearing methods as previously reported [4,5,6,7]. The timeline of each protocol showed the faster clearing process of the tDISCO method (Figure 2B). Next, we measured the tissue size before and after clearing. Our results showed that there was no significant difference in the degree of shrinkage between tDISCO and the other three methods (Figure 2C). We further analyzed the transparency of cleared samples. We found that the transparency of tDISCO-cleared brains did not significantly differ from the other three methods (Figure 2D). tDISCO provides a rapid and effective method for clearing the whole brain of adult mice.

### 2.3. tDISCO Can Be Applied to Clear Various Organs and Visualize Neuronal Morphology

**Figure 3:**
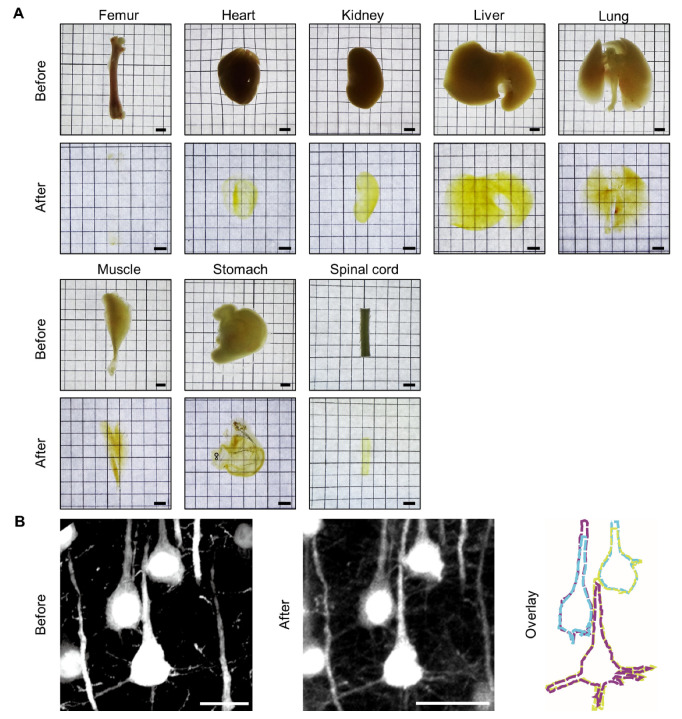
The clearing performance on different tissues/organs using tDISCO clearing. (**A**) Images of individual mouse organs before and after tDISCO clearing. (**B**) The neuronal morphology before and after tDISCO clearing. The outlines of three individual neurons before and after clearing were matched, the yellow, blue, and magenta colors (left) versus blue, magenta, and yellow colors (middle), separately. Scale bars: (**A**), 2 mm; (**B**), 20 μm.

In addition to its application on whole brains, tDISCO showed effective clearing capacity for various organs, such as hard bone (femur), internal organs (heart, kidney, liver, lung, and stomach), and muscle tissue (gastrocnemius muscle) (Figure 3A). In order to achieve a higher degree of transparency on heme-rich tissues (such as the heart and liver), a decolorization procedure was performed before tDISCO clearing (Table 1).

In order to evaluate the preservation of neuronal morphology after tDISCO clearing, the images of EGFP^+^ corticospinal neurons (CSNs) were captured using a confocal microscope before and after clearing. Our results showed that the outlines of neurons were sufficiently matched before and after tDISCO clearing (yellow, blue, and magenta colors before versus blue, magenta, and yellow colors after clearing; the structure similarity index, 0.969 ± 0.003) (Figure 3B). 

These data indicated the adequate clearing capacity of tDISCO on other various organs and maintenance of neuronal morphology after clearing.

### 2.4. tDISCO Allows 3D Visualization of Supraspinal Neurons with High-Resolution

**Figure 4:**
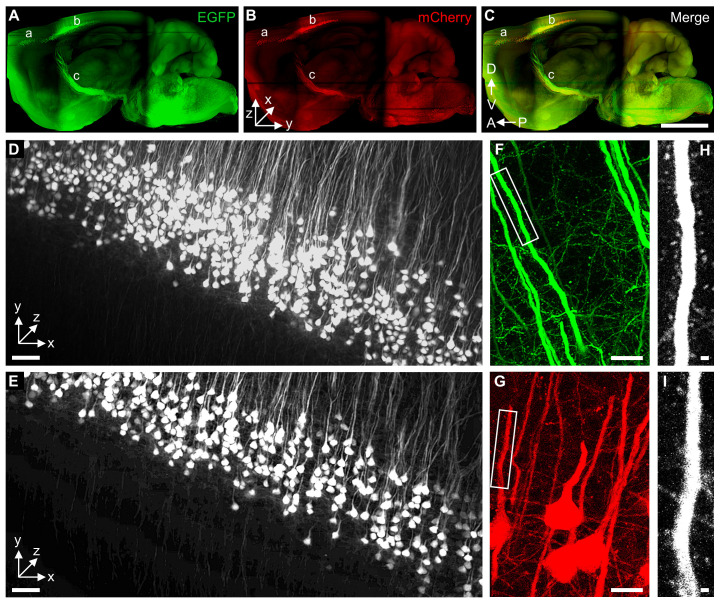
Three-dimensional visualization of AAV-labeled supraspinal neurons projecting to the cervical spinal cord. (**A**–**C**) Lateral view of the 3D reconstruction of supraspinal neurons and tracts labeled by AAV2-Retro. A-P: anterior to posterior; D-V: dorsal to ventral. The images were acquired with a Z.1 light-sheet microscopy (Zeiss, Germany) equipped with a 5×/0.16 Plan Neofluar objective at a zoom of 0.4. (**D**,**E**) The magnification images of EGFP^+^ (**D**) or mCherry^+^ (**E**) corticospinal neurons (CSNs) in the cortex. The images were acquired with a Z.1 light-sheet microscopy equipped with a 5×/0.16 Plan Neofluar objective at a zoom of 2.5. (**F**–**I**) High-resolution confocal imaging of 2 mm thick cleared brain slices using an LSM700 equipped with a C-Apochromat 40×/1.20 water objective at a zoom of 2.0. Representative images of EGFP^+^ (**F**,**H**) and mCherry^+^ (**G**,**I**) are shown. a, forelimb area; b, the central population in the sensorimotor cortex; c, the corticospinal tract. D-V: dorsal to ventral; A-P: anterior to posterior. Scale bars: (**A**–**C**), 2 mm; (**D**,**E**), 50 μm; (**F**,**G**), 10 μm; (**H**,**I**), 1 μm.

Global connectivity of supraspinal neurons and their axons can be depicted with retrogradely transducing AAV virus [25,26]. In order to test whether tDISCO can be used to visualize the whole brain labeled by AAV with EGFP and mCherry fluorescence proteins, AAV2-Retro-EGFP/mCherry was injected into the cervical spinal cord separately. Then, the brains were cleared by the tDISCO method. A series of images covering the whole brain was captured using light-sheet fluorescence microscopy. The supraspinal neurons located in the cortex, red nucleus, and reticular formation were labeled with EGFP and mCherry (Figure 4A–C). Consistent with previous studies of cervical projection CSNs [26,27], the EGFP^+^ and mCherry^+^ CSNs are located in separate areas in the cortex (Figure 4A–C): the rostral forelimb area (secondary motor cortex; Figure 4A–C a) and the central population (primary sensorimotor cortex; Figure 4A–C b). In addition to cell bodies, the axons of the corticospinal tract (CST) could be distinguished as they formed a unified tract before descending into the ventral medulla (Figure 4A–C c). High-resolution LSFM images in the cortex could be used to easily identify the structure of CSNs (Figure 4D,E and Supplementary Video S1). In order to image the dendritic spines of CSNs, the cleared whole brains were sliced into 2 mm thick coronal sections. The images were acquired using a confocal microscope equipped with a 40× objective (NA, 1.20) at a zoom of 2.0. In the high-resolution images in the cortex, the EGFP^+^ and mCherry^+^ dendritic spines could be reliably identified and reconstructed in 3D (Figure 4F–I). In order to test the preservation of mCherry fluorescence after tDISCO clearing, the cleared brain slice was imaged on day 4 and day 37. The merged image showed that the morphology of CSNs was well maintained, which is consistent with the matched outlines of EGFP^+^ CSNs mentioned above. The fluorescence intensity showed only a minor loss of mCherry signal on day 37 after tDISCO clearing (Supplementary Figure S3).

**Figure 5:**
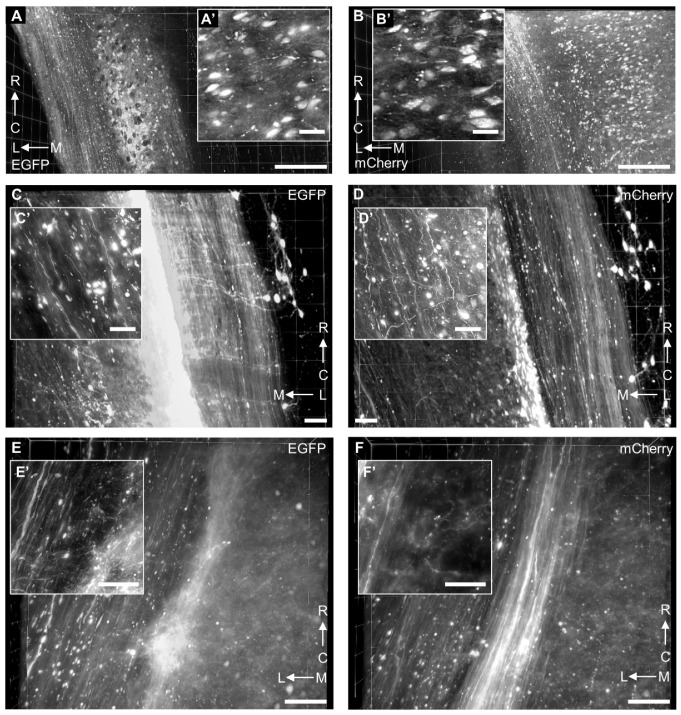
Three-dimensional visualization of AAV-labeled supraspinal axons in the spinal cord. (**A**,**B**) The representative image of EGFP^+^ and mCherry^+^ spinal interneurons located at the corresponding injection sites. (**A’**,**B’**) The high magnification of spinal interneurons captured by LSFM. The images were acquired with a Z.1 light-sheet microscopy (Zeiss, Germany) equipped with a 20×/1.0 Plan Neofluar objective at a zoom of 1.1. (**C**,**D**) The EGFP^+^ and mCherry^+^ supraspinal axons and spinal interneurons in the cervical spinal cord rostral to the injection site. (**C’**,**D’**) The high magnification image of EGFP^+^ and mCherry^+^ single axons in the gray matter captured by LSFM. (**E**,**F**) The EGFP^+^ and mCherry^+^ supraspinal axons in the middle thoracic spinal cord. (**E’**,**F’**) The high magnification image of EGFP^+^ and mCherry^+^ single axons in the gray matter of the middle thoracic spinal cord. M-L: midline to lateral; R-C: rostral to caudal. Scale bars: (**A**,**B**), 200 μm; (**C**–**F**), 50 μm; (**A’**–**F’**), 25 μm.

In order to detect the axons of supraspinal neurons at spinal levels, the cervical and thoracic spinal cords were cleared by tDISCO. The high-resolution images were captured by LSFM equipped with a 20× objective (NA, 1.0). The EGFP^+^ and mCherry^+^ spinal interneurons were observed in injection sites (Figure 5A,B). The high magnification images showed interneurons in single-cell resolution (Figure 5A’,B’). The supraspinal axons in white matter and their collateral branches in gray matter were also observed in the cervical spinal cords rostral to the injection site (Figure 5C,D,C’,D’) and middle thoracic spinal cords (Figure 5E,F,E’,F’).

Overall, tDISCO is appliable for 3D imaging and reconstruction of long projection neuronal circuits with high resolution.

## 3. Discussion

The organic solvent-based tissue clearing methods provide the rapid dehydration process and tissue shrinkage, which allow the imaging of larger samples [3,14]. In this study, we provided a fast and simple clearing protocol named tDISCO. The tDISCO protocol could preserve endogenous EGFP fluorescence for months. Then, we confirmed the performance of tDISCO on the adult mouse brain, including the degree of shrinkage and transparency and the maintenance of neuronal morphology. In addition to brain tissue, tDISCO showed a powerful clearing capacity for other tissues, such as the spinal cord, internal organs (liver, lung, stomach, kidney, and heart), and even hard tissue (femur). We further confirmed its application in the 3D visualization of EGFP^+^ and mCherry^+^ cervical projection CSNs and their dendritic spines labeled with the AAV virus.

The denaturation of GFP fluorescent protein in acid conditions and concentration of peroxide contributed to GFP quenching [20,28]. The alkaline pH conditions and lower temperature can extend the preservation of GFP fluorescence, as previous studies reported [5,24]. However, the dehydration process using *tert*-butanol was slower and more temperature-dependent than that using an a-THF solution in tDISCO for adult mouse brains (4 days, 35 °C versus 2.125 days, RT), which could explain the higher initial level of fluorescence. Although previous THF-based clearing methods, such as FDISCO [5] and sDISCO [19], showed fast clearing procedures, the complex chromatography processes to remove peroxides are necessary, which can be an obstacle for beginners to transparentize a sample. In this study, we found that the concentration of peroxides in the THF solution was always maintained at a low level within one year by using a long glass pipette for transferring the solution, which minimizes the air exposure of THF. Thus, we simplified the protocol of tDISCO by omitting the process for the purification of clearing solutions. 

The tissue shrinkage is induced during solvent-based clearing methods [4,7]. It is advantageous for large samples, especially when using Z.1 light-sheet microscopy, in which the size of the observation chamber is fixed [29]. Moreover, its chamber consists of several plastic screws that can be eroded in a-BABB-D4 solution, including DBE and BABB, during the long imaging process. In order to solve this trouble, ethyl cinnamate was used to fill the chamber in this study. Another solution is to use a different type of light-sheet microscopy. As previously reported, when using Ultramicroscope, the final RI-matching solutions (DBE or BABB) could be directly filled into the reservoir of the sample holder [4,17]. Since the spatial connectivity of supraspinal tracts and neuronal morphology were preserved well after tDISCO clearing, the reduced tissue size is more applicable for large samples during the imaging process. 

The remaining red material in cleared tissues, such as the liver, kidney, and heart, was reported using solvent-based clearing methods [5,7]. This dark color decreased tissue transparency and thereby limited the deep of imaging in large tissue. In order to solve this issue, the complete perfusion process is important before the tissue collection. We additionally treated these fixed tissues in 5% H_2_O_2_ solution before tDISCO clearing, which mostly alleviated the residual red color and increased the transparency of cleared liver, kidney, and heart. Although the treatment with hydrogen peroxide allows a higher degree of transparency on heme-rich tissues, the step would remove fluorescence as well. Another regent for decolorization, such as N,N,N′,N′-Tetrakis (2-hydroxypropyl) ethylenediamine, can be used [8]. 

The endogenous fluorescence proteins, such as EGFP and mCherry, transduced with AAV have been widely used to illuminate neural circuits and long projection neurons [26]. To compensate for the resolution limitation of light-sheet fluorescence microscopy, we combined a confocal microscope and cleared brain slices for imaging tiny structures, such as dendritic spines that can be reliably identified in tDISCO-cleared brains. The tDISCO method can be applied for high-resolution imaging of AAV-transduced endogenous fluorescence in the brain and spinal cord.

Although the fluorophore of mCherry is less sensitive to pH than GFP, its fluorescence intensity is higher in alkaline pH conditions [30]. Our data also showed that tDISCO could preserve the mCherry fluorescence over a month. However, considering the mCherry was derived from DsRed of coral *Discosoma*, unlike GFP variants [31], the optimal conditions for mCherry preservation should be further investigated. Moreover, more fluorescent proteins and chemical tracers should be further quantitatively tested. Like other solvent-based clearing methods, tDISCO is still incompatible with lipophilic dyes. Another limitation is the challenge of immunostaining on whole-mount tissue, and thereby tDISCO can only be applied to samples with fluorescence, such as GFP transgenic animals and virus transduction.

## 4. Materials and Methods

### 4.1. Animals

Wild-type C57BL/6 J mice (female, 8 weeks old, 18–22 g) were used in this study and ordered from CLEA Japan, Inc. The experiments were approved by the Laboratory Animal Center of Keio University School of Medicine (No: 19022) and were gently conducted by the Guide for the Care and Use of Laboratory Animals (NIH, Bethesda, MD, USA). The mice were housed in groups of five animals per cage in a 12 h light/dark cycle and ad libitum supply of standard chow and water environment (temperature- and humidity-controlled).

### 4.2. Retrograde Tracing of Supraspinal Inputs to the Cervical Spinal Cord

Adeno-associated virus (AAV) retrograde serotype, AAV2-Retro-hSyn-EGFP (cat. No. 50465; titer: ≥7 × 10^12^ vg/mL) and AAV2-Retro-hSyn-mCherry (cat. No. 114472; titer: ≥7 × 10^12^ vg/mL), were purchased from Addgene. All animals were anesthetized by intraperitoneal (i.p.) injection of ketamine (60 mg/kg) and xylazine (10 mg/kg). The laminal plates of C4/5 vertebrae were removed. Then, AAV2-Retro-EGFP (500 nL/site) was injected into the right spinal cord at 0.5 mm lateral to the midline and a depth of 0.7 mm [32,33]. AAV2-Retro -mCherry (500 nL/site) was injected into the left cord using the same method. After surgery, mice were kept on a heating pad (38 °C) until fully recovered. All experiments were conducted in effort to minimize animal suffering.

### 4.3. Tissue Processing and Imaging

After deep anesthesia using i.p. injection of ketamine (60 mg/kg) and xylazine (10 mg/kg), animals were transcardially perfused with cold saline and 4% paraformaldehyde (PFA). The internal organs (heart, kidneys, livers, lungs, and stomach), brains, spinal cords, gastrocnemius, and femurs were collected. All tissues were fixed in 4% PFA at 4 °C overnight. Then, tissues were stored at 0.05%NaN3/PBS solutions at 4 °C. One-millimeter-thick brain slices were collected using a vibratome (VT1200s, Leica, Wetzlar, Germany).

### 4.4. Modified Organic Solvent-Based Tissue Clearing Method

The tDISCO clearing procedure was modified in three ways to make its protocol more user-friendly: (1). no requirement of complex apparatus for removing peroxides in THF; (2). alkaline pH condition (pH 9.0~10.0) in THF solutions(a-THF) contributing to fluorescence preservation; (3). replacement of DBE solution with a-BABB-D4 (alkaline pH of BABB-D4, pH 9.0~10.0). In order to test the peroxide level in old THF and DBE stocks, its concentration was measured using Quantofix peroxide test strips (Sigma-Aldrich, cat. No. Z249254-1PAK, St. Louis, MO, USA). The dura and other fascia tissues on the organs, such as brains and spinal cords, were carefully removed under a stereo microscope (SZ61, Olympus, Tokyo, Japan) before clearing. 

In order to avoid air exposure to THF solution, the solution was gently transferred using disposable glass Pasteur pipettes connected with a rubber head (CORNING, cat. No. 7095D-9) and sealed tightly using parafilm (Amcor, cat. No. PM996) after usage. The fixed samples were completely immersed in a series concentration of a-THF/dH_2_O solutions (Table 1; 50%, 70%, 80%, 100%; Sigma-Aldrich, cat. No. 401757). After dehydration and delipidation, samples were transferred to the a-BABB-D4 solution (alkaline pH of BABB-D4, pH 9.0~10.0), which is the mixture of BABB (benzyl alcohol + benzyl benzoate at a ratio of 1:2 in volume, Sigma, cat. No. 305197, and Nacalai, cat. No. 04601-65) and diphenyl ether (DPE, Nacalai, cat. No. 13804-35) at a ratio of 4:1 in volume and adding 0.4 *v*/*v*% vitamin E (Nacalai, cat. No. 34114-12). The pH of all solutions was adjusted to pH 9.0~10.0 with triethylamine (Nacalai, cat. No. 34805-75). Samples and solutions were sealed into glass vials and placed on a rotating wheel at room temperature (RT). For better preservation of fluorescence in the long term, the cleared samples were stored in a-BABB-D4 solution at 4℃ and covered with alumina foil. For decolorization of the liver, kidney, and heart before clearing, fixed organs were bleached in 5% hydrogen peroxide solution (H_2_O_2_) in methanol at 4 °C overnight in a fume hood. In order to decalcify the bone tissues, the fixed femurs were immersed in the decalcification solution (10 *w*/*v*% EDTA in 0.01 M PBS, pH 8~9) for 3 days at 37 °C with gentle shaking before clearing [22].

### 4.5. Other Organic Solvent-Based Tissue Clearing Methods

For comparison, the 3DISCO, uDISCO, and FluoClearBABB methods were performed according to their original papers [4,6,7]. Briefly, in the 3DISCO clearing group, the fixed samples were put in glass vials and successively immersed in a series of THF/dH_2_O solutions for 12 h/step (50%, 70%, 80%, and third times 100%). Then, to homogenize the refractive index of whole samples, they were transferred to fresh DBE (25 g, Nacalai, 04618-52; 1L, Sigma-Aldrich, cat. No. 108014-1KG) for 12 h before imaging. The glass vials were placed on a rotating wheel at RT during all processes. In the uDISCO group, the fixed samples were immersed in a series of *tert*-butanol/dH_2_O solutions (30%, 50%, 70%, 80%, 90%, 96%, and 100%, Nacalai, 11714-75) at 35 °C, then transferred to dichloromethane solution (DCM; Sigma-Aldrich, 270997) for 1 h RT, finally transferred to BABB-D4 solution for 10 h. In the FluoClearBABB group, the fixed samples were immersed in a series of *tert*-butanol/dH_2_O solutions (30%, 50%, 70%, 80%, 96%, and twice 100%, pH 9.5) for 24 h/step at 30 °C, then transferred to BABB (the mixture of benzyl alcohol and benzyl benzoate at a ratio of 1:2 in volume, Sigma, cat. No. 305197, and Nacalai, cat. No. 04601-65) for 48 h.

### 4.6. Measurement of Transparency and Tissue Size

The transparency and shrinkage size before and after clearing were measured as previously described [34]. Briefly, images of whole brains were captured before and after clearing using a stereo microscope (SZX16, Olympus, Japan) equipped with KL 1600 LED. Horizontal images from two focal planes were acquired. The first plane was adjusted to measure the brain’s dimension (Supplementary Figure S4A). The second plane was adjusted to the plane of a background grid placed under the sample (Supplementary Figure S4B). The average pixel intensities from four adjacent rectangular regions of interest (ROIs) within light vs. dark areas of the grid were calculated using Fiji (NIH, USA), which were used to estimate the average Michelson contrast (C) using the following equation:

(1) $$C=\frac{I_{max}-I_{min}}{I_{max}+I_{min}}$$

*I_max_* is the maximum intensity from light areas and *I_min_* is the minimum intensity from dark areas.

Then, the transparency (*t*) of the individual brain was calculated with the following equation:

(2) $$t=\frac{C_{brain}}{C_{ctrl}}$$

where *C_brain_* is the contrast of the grid below the cleared brain and *C_ctrl_* is the contrast outside the cleared brain.

In order to estimate shrinkage, the below equation was used.

(3) $$R=\frac{D_{post}}{D_{pre}}$$

The full width of the brain from the left edge to the right edge was measured.

Where *D_post_* is the brain length obtained after clearing, and *D_pre_* is the brain length obtained before clearing.

### 4.7. Confocal Fluorescence Microscopy Imaging

For quantitative analysis of fluorescence signals after tDISCO, 3DISCO, uDISCO, and FluoClearBABB clearing, three z-stack images (depth, 1.62 μm; thickness, 81 μm) per slice were captured using LSM700 confocal microscope (Zeiss, Jena, Germany) equipped with a Plan-Apochromat 20×/0.8 M27 dry objective. The same area in the cortex was captured for each slice over time, which was kept consistent for each method. The MIP of z-stack was performed using Z projection function in Fiji. The fluorescence level quantification was calculated using Fiji at 0, 4, 7, 14, 21, 28, and 56 days after clearing. We used the default threshold function of Fiji software to identify the EGFP signal. Then, the mean intensity of each MIP image was measured.

In order to compare neuronal morphology before and after tDISCO clearing, three z-stack images of 1 mm thick brain slices were captured using LSM700. The neuronal morphology before and after clearing was outlined separately using Adobe Illustrator (Adobe Inc., San Jose, CA, USA). The structure similarity index for neurons was calculated as previously described [35]. The index was measured using an MS-SSIM plugin installed in Fiji (NIH, USA).

The cleared whole brains were imaged by LSFM. Then, they were sliced into 2 mm thick coronal sections using a FEATHER Disposable Scalpel (NO.11, FEATHER Safety Razor Co., Ltd., Osaka, Japan). The z-stack images of dendritic spines (depth, 0.467 μm; thickness, 7.4 μm) in the cortex were captured from the coronal sections with an LSM700 confocal microscope equipped with a C-Apochromat 40×/1.20 water objective at a zoom of 2.0.

### 4.8. Light-Sheet Fluorescence Microscopy (LSFM) Imaging

When samples became transparent, images were captured using Z.1 light-sheet microscopy (Z.1, Zeiss, Jena, Germany) equipped with a 5×/0.16 Plan Neofluar objective at a zoom of 2.5 and a 20×/1.0 Plan Neofluar objective at a zoom of 1.1. The cleared samples were transferred to a self-made adapter (ferromagnetic feature) attached to a tip-cut 1 mL syringe body (Terumo, Tokyo, Japan) with a 5.0 mm magnet fixed on the edge. Then, the samples were fully immersed in ethyl cinnamate (Eci, Sigma-Aldrich, 112372-100G) in the observation chamber rather than the final RI-matching solution (a-BABB-D4) because several plastic screws on the observation chamber can be eroded in a-BABB-D4 solution during the imaging process. 

In order to image the whole brain, tile scans were used to cover the entire sample. The row image data were tiled using Zen software (Zeiss, Germany). Next, 3D reconstructions were performed using the ClearVolume [36] plugins installed in Fiji [37]. Autofluorescence was removed using the Background Subtraction function, and movies were generated using the ClearVolume plugins and merged in Photoshop CS6 (Adobe Inc., San Jose, CA, USA).

### 4.9. Statistical Analysis

All experimental data were presented as the mean ± SD and analyzed using Fiji (NIH, USA) and GraphPad Prism 8.0 (GraphPad Inc., San Diego, CA, USA). A total of three brain slices for each method were analyzed, and five whole brains per method were used. Two-way ANOVA with Tukey’s multiple comparisons was used for fluorescence preservation, and one-way ANOVA with Tukey’s multiple comparisons was used for transparency and tissue shrinkage. Significant differences were defined at * *p* < 0.05, ** *p* < 0.01, *** *p* < 0.001, and n.s., not significant *p* > 0.05.

## 5. Conclusions

In this study, we provide a fast and simple clearing method, tDISCO, which can preserve AAV-transduced endogenous EGFP fluorescence for months. It can be further applied for 3D imaging and reconstruction of neural circuits and long projection neurons with high resolution.

## Figures and Tables

**Table 1:** Clearing protocols for different samples.

Reagents (Alkaline pH)	Brain Slices (1 mm); Spinal Cord	Brain Hemisphere; Internal Organs ^a^	Whole Adult Brain
50% (*v*/*v*) a-THF	30 min	3 h	6 h
70% (*v*/*v*) a-THF	-	-	6 h
80% (*v*/*v*) a-THF	30 min	3 h	12 h
100% (*v*/*v*) a-THF	2 × 30 min	3 h, ON	2 × 12 h
a-BABB-D4	≥15 min	≥1.5 h	≥3 h

^a^ liver, kidney, and heart (internal organs) were bleached in 5% hydrogen peroxide solution (H_2_O_2_) in methanol at 4 °C overnight before tDISCO clearing. To optimize the clearing protocol for different tissues, the concentration of a-THF solutions can be further modified by adding 30% or 90%.

## Data Availability

The data that supporting this study’s findings are available from the corresponding authors upon reasonable request.

## Supplementary Materials

The following supporting information can be downloaded at: https://www.mdpi.com/article/10.3390/ijms23179637/s1.
