# Supplementary material for: Long Preservation of AAV-Transduced Fluorescence by a Modified Organic Solvent-Based Clearing Method
Source: Int J Mol Sci. 2022 Aug 25;23(17):9637. doi: 10.3390/ijms23179637 (PMC9455935; doi:10.3390/ijms23179637)
Supplement: Supplementary file 1 [file ijms-23-09637-s001.zip › Supplementary_Material 220825.pdf]

Supplementary_Material

Title: Long preservation of AAV-transduced fluorescence by a modified organic solvent-based clearing method

Authors: Tao Lu[1], Munehisa Shinozaki[1], Narihito Nagoshi[2], Masaya Nakamura[2]*, and Hideyuki Okano[1]*

Affiliations: [1]Department of Physiology, Keio University School of Medicine, 35 Shinanomachi, Shinjuku-ku, Tokyo 160-8582, Japan.

[2]Department of Orthopaedic Surgery, Keio University School of Medicine, 35 Shinanomachi, Shinjuku-ku, Tokyo 160-8582, Japan.

***Co-corresponding Authors:** Masaya Nakamura (masa@keio.jp) and Hideyuki Okano (hidokano@keio.jp)

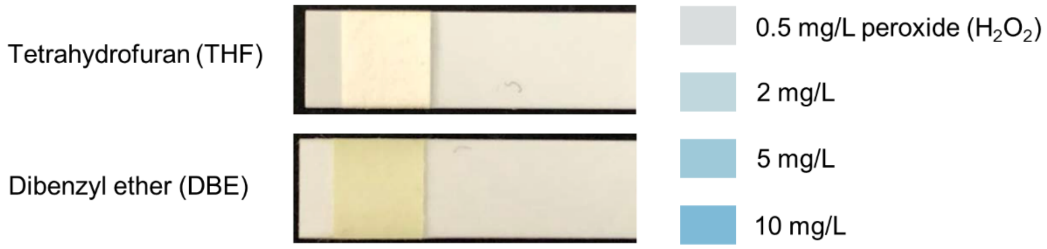

Supplementary Figure S1. The concentration of peroxide in tetrahydrofuran (THF) and dibenzyl ether (DBE). The deeper color means a higher concentration of peroxide.

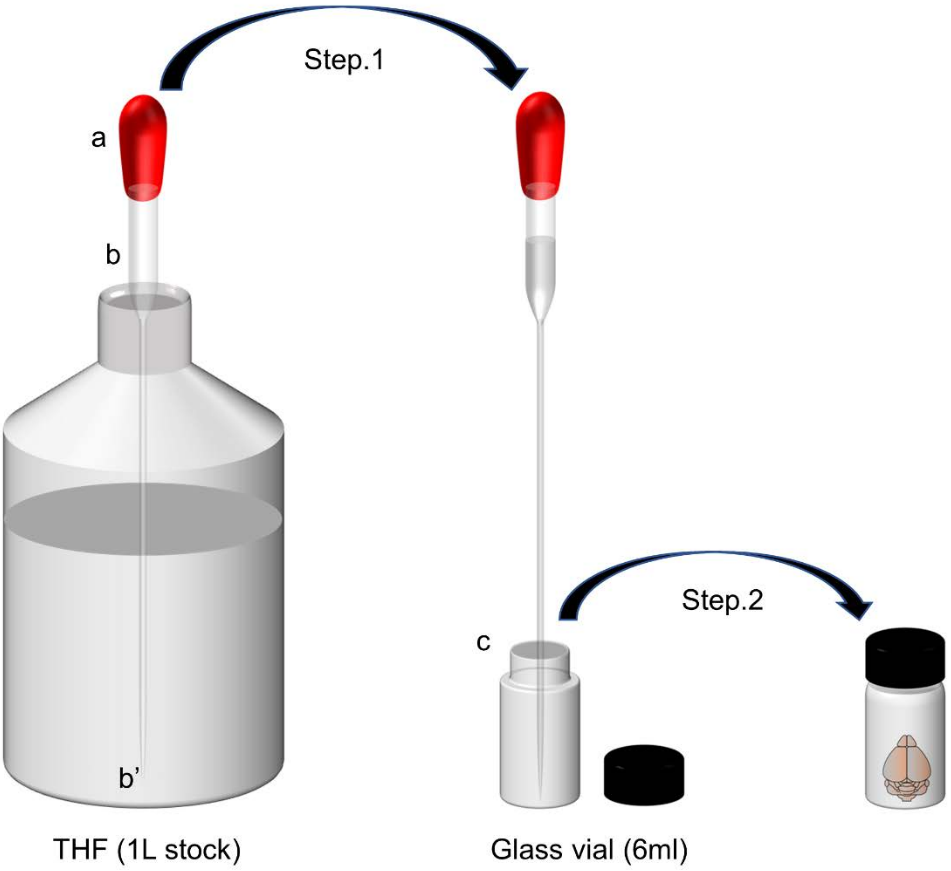

Supplementary Figure S2. The schema for transferring tetrahydrofuran (THF) using a long glass pipette (step.1). The THF solution in the bottom was used each time. The glass vial was fully filled, then, tightly sealed (step.2) before placing on a rotating wheel. a. a rubber head; b. a glass pipette; b’. the tip of a glass pipette; c. a glass vial.

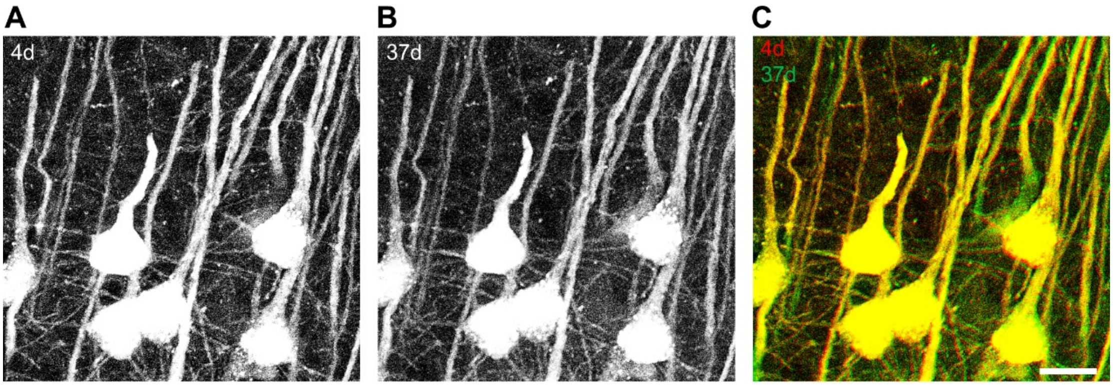

Supplementary Figure S3. Long-term preservation of AAV-labeled mCherry endogenous fluorescence. A. The representative images of $mCherry^{+}$ corticospinal neurons at day 4 after tDISCO clearing. B. The representative images of $mCherry^{+}$ corticospinal neurons at day 37 after tDISCO clearing. C. the merged images of day 4 (red) and day 37 (green) mCherry fluorescence. The images were captured with an LSM700 equipped with a C-Apochromat 40x/1.20 water objective at a zoom of 2.0 from 2-mm thick cleared brain slices. Scale bar: 10 µm.

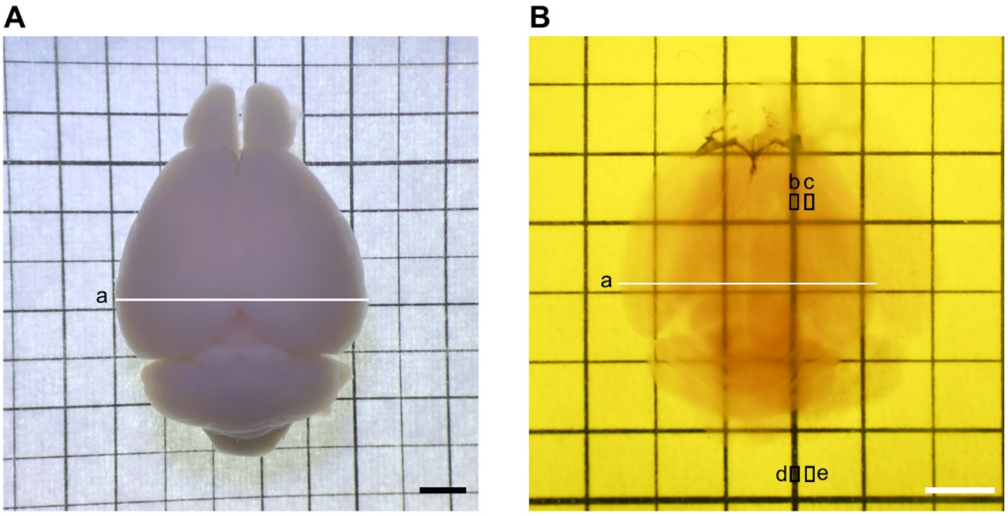

Supplementary Figure S4. Illustration of a measurement method for transparency and tissue shrinkage. A. The representative image of the first plane for adult mouse brain before tDISCO clearing (illumination from below). a, the white line shows the calculated width from the left to the right edge. B. The representative image of the second plane for adult mouse brain after tDISCO clearing (illumination from above). a, the white line shows the calculated width from the left to the right edge. b and c, ROIs used to calculate contrast within the cleared brain; d and e, ROIs used to calculate contrast outside the cleared brain. Scale bar: 2 mm.

Supplementary Video S1. 3D reconstruction of the dendrites and spines of corticospinal neurons labelled by AAV2-Retro after tDISCO clearing. After whole-brain imaging by LSFM, the brains were sliced into 2-mm thick coronal sections. The z-stack images of dendritic spines in the cortex were captured from the coronal sections with an LSM700 confocal microscope equipped with a C-Apochromat 40x/1.20 water objective at a zoom of 2.0. The videos were generated using ClearVolume plugins in Fiji and merged in Photoshop CS6.
